# Therapy with recombinant human antithrombin, heparin and tissue plasminogen activator improves survival and reduces ventilation days in a long-term ovine model of cutaneous burn and smoke inhalation injury

**DOI:** 10.1186/cc9600

**Published:** 2011-03-11

**Authors:** S Asmussen, Y Yamamoto, DL Traber, H Ito, R Cox, H Hawkins, LD Traber, D Herndon, P Enkhbaatar

**Affiliations:** 1 University of Texas Medical Branch, Galveston, TX, USA; 2 Shriners Hospital for Children, Galveston, TX, USA

## Introduction

In this study we investigated the long-term effects of a combined therapy with recombinant human antithrombin (rhAT), heparin (hep) and tissue plasminogen activator (tPA) in our established model of acute lung injury, resulting from burn and smoke inhalation injury (BSII). We hypothesised that this triple therapy decreases the requirement of ventilation, reduces ventilation days and improves survival.

## Methods

Ten female sheep (34.4 ± 2.1 kg) were operatively prepared for chronic study, and were randomly allocated either to control or treatment groups (*n *= 5 each). After tracheostomy, BSII (48 breaths of cotton smoke) and third-degree burn of 40% total body surface area was performed under deep anesthesia. The sheep were mechanically ventilated and fluid resuscitated for 96 hours in an awake state. The therapy group received combined therapy of rhAT, nebulized heparin and nebulized tPA. The continuous i.v. infusion of 0.7 mg/kg/hour rhAT was started 1 hour post-injury. The nebulizations of 5,000 IE heparin every 4 hours were started 2 hours post-injury and 2 mg tPA were nebulized every 4 hours, starting 4 hours post-injury. The treatment was stopped at 48 hours. Ventilator weaning was started at 48 hours, if PaO_2_/FiO_2 _ratio ≥250. The control group received saline nebulization. Measurements were taken in intervals ranging from 3 to 12 hours. Statistical analysis: two-way ANOVA and Bonferroni *post-hoc *comparison. Data are expressed as mean ± SEM. Significance *P *< 0.05.

## Results

The PaO_2_/FiO_2 _ratio was significantly decreased in the control group versus baseline (BL: 530 ± 16 vs. 96 hours: 267 ± 51). The ratio showed significantly higher values in the treatment versus control sheep (96 hours: 377 ± 32). All treated sheep survived and were weaned from the ventilator. Four out of five treatment sheep could be decannulated from the tracheostomy tube at 72 hours. Only three out of five control sheep survived 96 hours and none of the control sheep could be weaned from the ventilator.

## Conclusions

This triple therapy with nebulization of heparin and tPA and intravenous application of rhAT may be a novel and efficient therapeutic alternative to improve the outcome of burn patients with smoke inhalation injury.

